# A Loss Differentiation Method Based on Heterogeneous Ensemble Learning Model for Low Earth Orbit Satellite Networks

**DOI:** 10.3390/e25121642

**Published:** 2023-12-10

**Authors:** Debin Wei, Chuanqi Guo, Li Yang, Yongqiang Xu

**Affiliations:** 1 Communication and Network Laboratory, College of Information Engineering, Dalian University, Dalian 116622, China; guochuanqi@s.dlu.edu.cn (C.G.); xuyongqiang@s.dlu.edu.cn (Y.X.); 2 School of Automation, Nanjing University of Science and Technology, Nanjing 210094, China

**Keywords:** LEO satellite networks, loss differentiation, ensemble strategy, heterogeneous ensemble learning

## Abstract

In light of the high bit error rate in satellite network links, the traditional transmission control protocol (TCP) fails to distinguish between congestion and wireless losses, and existing loss differentiation methods lack heterogeneous ensemble learning models, especially feature selection for loss differentiation, individual classifier selection methods, effective ensemble strategies, etc. A loss differentiation method based on heterogeneous ensemble learning (LDM-HEL) for low-Earth-orbit (LEO) satellite networks is proposed. This method utilizes the Relief and mutual information algorithms for selecting loss differentiation features and employs the least-squares support vector machine, decision tree, logistic regression, and K-nearest neighbor as individual learners. An ensemble strategy is designed using the stochastic gradient descent method to optimize the weights of individual learners. Simulation results demonstrate that the proposed LDM-HEL achieves higher accuracy rate, recall rate, and F1-score in the simulation scenario, and significantly improves throughput performance when applied to TCP. Compared with the integrated model LDM-satellite, the above indexes can be improved by 4.37%, 4.55%, 4.87%, and 9.28%, respectively.

## 1. Introduction

Satellite communication has the advantages of long communication distance, wide coverage, and no geographical restrictions, and plays an irreplaceable role in current and future communication systems [1]. Satellite networks operate in a wireless environment that is highly susceptible to solar activity, cosmic particle radiation, noise interference, etc., resulting in a bit error rate ranging from 10^−8^ to 10^−5^. Moreover, the Earth’s atmosphere, ionosphere, and ice crystal rainfall layer also affect satellite-to-ground links, leading to higher bit error rates, especially in military scenarios where human interference can cause even higher rates exceeding 10^−5^. In traditional TCP, packet loss is considered as an indication of network congestion by default. Therefore, the congestion avoidance mechanism needs to be enabled, which reduces the congestion window. However, in satellite networks, apart from packet losses caused by congestion, it is crucial not to ignore packet losses caused by channel errors. If network congestion is the default, the congestion window will be reduced, resulting in inefficient utilization of network bandwidth and compromising network reliability. 

In light of these issues, extensive research has been conducted by numerous scholars [2,3]. From a measurement acquisition perspective, packet loss differentiation can be categorized into explicit and implicit methods. Explicit loss differentiation primarily relies on the active queue management mechanism of routing nodes to determine the cache queue occupancy and provide feedback to the sender. This approach is more direct and accurate but requires support from intermediate nodes. Implicit loss differentiation mainly utilizes end-to-end round-trip time (RTT) [3], relative one-way trip time (ROTT) [4,5], packet inter-arrival time (IAT) [6], loss number [5], and other decision mechanisms to identify types of packet loss [7,8,9,10]. It does not require the support of nodes other than the sender and receiver, and can be implemented with minimal changes in the network.

Implicit loss differentiation can further be divided into threshold-based and learning algorithm-based loss differentiation methods. Packet loss differentiation based on threshold determination involves utilizing statistical analysis and calculation of feature parameter values, such as RTT, ROTT, IAT, etc., to characterize the network state. By comparing these feature parameters with predefined thresholds, the type of packet loss can be determined. Although this algorithm has low complexity, it heavily relies on experiential threshold settings. Additionally, relying solely on a single feature cannot achieve high determination accuracy and defining the relationship between multiple feature thresholds is challenging, which ultimately affects the accuracy of packet loss differentiation.

In machine learning-based packet loss discrimination algorithms, decisions are made using models that learn correlations between features. Among them, decision trees (DTs), random forests (RFs), artificial neural networks (ANNs), K-nearest neighbors (KNNs), naive Bayes (NB), and other single classification models have become the mainstream methods for packet loss differentiation. However, the accuracy of differentiation in these single classification models is significantly affected by the number and features of samples, and their accuracy may not reach optimal levels. 

Ensemble learning is a classification method that combines results from multiple individual classifiers to obtain a final result, typically achieving better classification performance than using a single classifier alone while also offering greater diversity compared to homogeneous integration methods. Based on literature research findings, existing loss differentiation algorithms lack feature selection methods, individual classifier selection strategies, and effective ensemble strategy for accurate packet loss differentiation. 

Therefore, we proposed a novel loss differentiation algorithm based on heterogeneous ensemble learning. The algorithm employs ROTT along with average ROTTs, ROTT deviations, and IAT statistics (including maximums, minimums, and averages) as features for loss differentiation. The Relief method, combined with mutual information techniques, is utilized for feature selection. Q statistics and double failure (DF) value are used for the selection of individual classifiers in ensemble learning, including least-squares support vector machine (LSSVM), DT, logistic regression (LR), and KNN algorithm. The integration strategy of the heterogeneous ensemble learning model is designed using the stochastic gradient descent (SGD) method, with dynamically adjusted voting weight vectors for each classifier to enhance classification accuracy.

The rest of this paper is organized as follows. We analyze the related works on loss differentiation algorithms from threshold determination-based loss differentiation and learning algorithm-based loss differentiation methods in Section 2. In Section 3, we employ the Relief and mutual information method to construct a feature set that distinguishes packet loss in LEO satellite networks. In Section 4, we propose a heterogeneous ensemble learning model for discriminating packet loss in satellite networks. In Section 5, the loss differentiation method based on heterogeneous ensemble learning (LDM-HEL), loss detection method (LDM)-satellite [11], and LLD method [12] are used as comparative experiments for simulation, which proves the superiority of the algorithm proposed in this paper. Finally, Section 6 concludes the paper.

## 2. Related Work

### 2.1. Threshold Determination-Based Loss Differentiation

In terms of threshold determination-based loss differentiation algorithms, the Biaz scheme [4] utilizes packet IAT to discriminate between types of packet loss. The mBiaz scheme [5] offers a tradeoff between low misclassification of congestion losses and high throughput by modifying the threshold in Biaz. The Spike scheme [6] determines link congestion by comparing the ROTT with two thresholds, while the ZigZag scheme [5] distinguishes packet loss based on the number of lost packets, mean ROTT, and deviation. Samaraweera [13] proposed an end-to-end non-congestion packet loss detection (NCPLD) algorithm that measures the sender’s RTT and compares it with measured delay without congestion to determine the type of packet loss. Jeyasekar et al. [14] proposed an RTT estimation method based on the ARIMA (2,1,1) model and calculated packet backlogs using estimated RTT mutations to differentiate between congestion losses and non-congestion losses. In [15], a loss differentiation method, RTT ECN loss differentiation (RELD), based on ECN signaling and RTT is proposed, taking into account the packet number of ECN tags, the number of lost packets, RTT mean, and RTT deviation. TCP-wireless environment, link losses, and congestion packet loss models (TCP-WELCOME) [16] distinguish packet loss by observing the history of RTT sample evolution on the link and the data packet loss trigger. The loss differentiation algorithm, loss recovery, and differentiation (TCP-LoRaD) [17] is modified based on the TCP-WELCOME algorithm to calculate RTT. TCP Westwood [7] relies on end-to-end bandwidth estimates to determine the cause of packet loss. The authors in [8] set different thresholds according to the amount of unconfirmed data in the network to determine the type of packet loss. Considering that link error packet loss in the network is related to packet size, and packet loss caused by congestion is independent of packet size, the authors of [18] proposed a WMPLD (Wireless Multimedia Packet Loss Discrimination) scheme. In [19], the WMPLD threshold is modified, and a WMPLD+ scheme is proposed; different simulation scenarios are designed to analyze the performance of Biaz, mBiaz, Spike, TFRC satellite, WMPLD, and WMPLD+ schemes.

Furthermore, a loss differentiation algorithm for long-term evolution (LDA-LTE) [9] is proposed in 4G-LTE mobile networks to analyze loss patterns in congestion and link error scenarios based on parameters such as congestion level, average packet loss rate, average RTT, number of continuous losses, frequency of losses, and transmission delay jitter. Differentiation-based opportunistic linked-increases algorithm (D-OLIA) [10] identifies packet loss types by combining characteristic values of delay jitter and congestion window jitter, thus addressing the limitation of relying solely on delay or congestion window analysis. However, these methods typically require setting thresholds for the features used in their algorithms. The determination of thresholds primarily relies on experience, and establishing the relationship between multiple thresholds proves challenging, thereby impacting the accuracy of loss differentiation.

### 2.2. Learning Algorithm-Based Loss Differentiation

With the continuous advancement of data mining and machine learning technologies, incorporating time characteristics of packet transmission into machine learning models for loss differentiation can significantly enhance the accuracy of loss differentiation and improve network performance. Liu et al. [20] proposed a packet loss classification technique based on the disparity in RTT measurements between wireless losses and congestion losses, leveraging the estimation capabilities of hidden Markov models for Gaussian components. In [21], the authors introduced a Bayesian packet loss detection mechanism that utilizes end-to-end RTT measurements. The simulation results demonstrate that this Bayesian detector achieved a detection probability exceeding 80% with a false alarm probability below 20%, leading to more than 25% improvement in network performance. The above mechanisms only apply one packet feature and the performance of packet loss differentiation is not optimal. 

Reference [22] constructed a naive Bayes discrimination model based on the statistics of the packet loss ratio of high- and low-priority packets, and packet time interval, so as to capture packet loss state and effectively classify wireless and wired packet loss types. Based on the queue length in the cache, a clustering method based on unsupervised learning was proposed in [23] to distinguish packet loss. Chen et al. [12] extracted state information such as RTT, RTT_min_, RTT_max_, RTT/RTT_min_, RTT/RTT_max_, (RTT − RTT_min_)/(RTT_max_ − RTT_min_), and CWND/CWND_max_ from flows where packet losses are detected at TCP receivers and employ them as inputs to a neural network model to establish a packet loss model. Reference [24] defined various features related to packet losses and employed SVM for distinguishing wired/wireless hybrid networks’ packet losses. Reference [25] employed the cuckoo search back-propagation neural network (CSBPNN) algorithm to effectively distinguish packet loss. Molia et al. [26] proposed a reinforcement learning-based loss differentiation (RLLD) algorithm to distinguish TCP packet loss into congestion loss, link error loss and route loss by combining RTT, ACK, and TCP socket information, and proposed a reinforcement learning-based TCP transmission control method (TCP-RLLD). The authors in [10] proposed LDM-satellite, which is a machine learning-based congestion control method capable of end-to-end packet loss discrimination and congestion control by constructing an integrated classifier comprising multiple decision trees along with naive Bayes classifier, while utilizing ACK header flag bits for result feedback. However, this paper does not specify the features of input data packets during the machine learning process. 

## 3. Loss Differentiation Feature Selection for LEO Satellite Network

Utilizing machine learning techniques to identify the causes of packet loss, our first task is to extract relevant features from the time information of packet transmission.

### 3.1. Loss Differentiation Features for LEO Satellite Network

The ROTT is defined as the time difference between sending a packet at the sender and receiving it at the receiver. Accurate time synchronization of end-to-end satellite nodes is required to obtain this parameter. In congested areas, routers experience an increase in ROTT due to a large number of packets being queued in the cache for forwarding. When incoming packets exceed the cache capacity, packet loss occurs as they cannot enter the cache. However, wireless loss exhibits a random state and does not significantly affect the ROTT. Therefore, analyzing ROTT is crucial for identifying the causes of packet loss in satellite networks. Table 1 presents features related to end-to-end ROTT.

The IAT refers to the duration between two adjacent packets arriving at the receiver and plays a vital role in Biaz and mBiaz schemes. These schemes consider that if packet IAT falls within a certain range, bit errors are likely causing packet loss. If a packet arrives much earlier than expected, previous packets may have been discarded from buffer memory; if it arrives much later than anticipated, queue delay might have increased in buffers. In either case, network congestion can be attributed as the cause of packet loss. Table 2 outlines features regarding IAT.

Furthermore, the concept of consecutive lost packets is introduced in Table 3.

In machine learning applications, data classification accuracy and efficiency heavily rely on feature selection methods. Filtering, encapsulation, and embedding are commonly used techniques for feature selection. The filtering method is independent of the classifier, but it scores the features based on the difference or correlation between sample data and selects features by setting a scoring threshold or specifying the number of features. Consequently, the feature subset can be determined prior to classification, demonstrating excellent adaptability. In this study, we combine Relief and mutual information methods for feature selection.

### 3.2. Packet Loss Differentiation Feature Selection Method

The Relief algorithm represents a typical filtering approach. Its feature scoring and selection process is decoupled from classification algorithms, making it simple, convenient, and low in complexity. However, it is only suitable for calculating binary classification feature weights. The underlying principle is as follows: Firstly, positive samples and negative samples are segregated within the training dataset. For any given sample in the training dataset, one nearest-neighbor sample is selected from both same-class and different-class samples. If a particular feature attribute present in that sample exhibits greater similarity with its nearest neighbor from the same class compared to its nearest neighbor from a different class, it indicates a higher differentiation capability of that feature attribute. Consequently, larger feature scores or weights are assigned. 

Let the training set be $D=\{(x_{i},y_{i})|x_{i}\in R^{m},m>0,y_{i}\in \{1,-1\},i=1,2,\cdots ,n\}$, where *n* is the number of samples in the training set, and *m* is the number of feature attributes contained in each sample. The sample is $x_{i}=(x_{i}^{1},x_{i}^{2},\cdots ,x_{i}^{k},\cdots ,x_{i}^{m})$. Define the difference between samples $x_{i}$ and $x_{j}$ on the *k*th attribute as shown in Equations (1) and (2).

If the feature is scalar:

(1) $$\mathrm{diff}(k,x_{i},x_{j})=\left\{ \begin{array}{l} 0,\;\;\;\;\;\;x_{i}^{k}=x_{j}^{k}\\ 1,\;\;\;\;\;\;x_{i}^{k}\neq x_{j}^{k} \end{array}\right.$$

If the feature is numeric:

(2) $$\mathrm{diff}(k,x_{i},x_{j})=\left|\frac{x_{i}^{k}-x_{j}^{k}}{\underset{1\leq l\leq n}{\max}\{x_{l}^{k}\}-\underset{1\leq l\leq n}{\min}\{x_{l}^{k}\}}\right|$$

If $x_{i,\mathrm{nh}}$ is the homogeneous nearest neighbor of $x_{i}$, and $x_{i,\mathrm{nm}}$ is the heterogeneous nearest neighbor of $x_{i}$, then the iterative calculation formula for the weight of the *k*th feature is as follows:

(3) $$\theta_{k}(i)=\theta_{k}(i-1)-\frac{\mathrm{diff}(k,x_{i},x_{\mathrm{nh}})}{n}+\frac{\mathrm{diff}(k,x_{i},x_{\mathrm{nm}})}{n},i=1,2,\cdots ,n$$

After calculation, let $\theta_{k}(n)=\theta_{k}$, and subsequently the larger $\theta_{k}$ denotes a stronger classification ability associated with the *k*th feature attribute. Each individual weight of each specific feature is calculated using Equation (3) to form a vector $\theta ={\left(\theta_{1},\theta_{2},\cdots ,\theta_{m}\right)}^{T}$ representing all respective weights. These weights can then be arranged in descending order to determine their relative importance as well as establish corresponding arrangement order for each respective featured attribute. This determines their overall significance. 

The Relief algorithm solely ranks the importance of featured attributes without reflecting correlations among them. Therefore, we utilized the mutual information algorithm to analyze feature correlations, aiming to reduce redundancy between feature attributes and optimize the final feature selection results.

The concept of information entropy quantifies the level of uncertainty associated with the occurrence of each possible event within an information source, which is defined as:

(4) $$H(X)=-\sum_{i}p(x_{i})\times \log_{2}p(x_{i})$$

where $p(x_{i})$ is the frequency of event $x_{i}$. $X=\{x_{1},x_{2},\cdots ,x_{n}\}$ is the information source containing all possible states.

The conditional information entropy of *X* with respect to *Y* quantifies the additional knowledge provided by *X* when *Y* is known for a pair of associated random variables:

(5) $$H(X|Y)=-\sum_{i,j}p(x_{i},y_{j})\times \log_{2}p(x_{i}|y_{j})$$

where $Y=\{y_{1},y_{2},\cdots ,y_{n}\}$, $p(x_{i},y_{j})$ is the joint probability density function of the random variable (X,Y), and $p(x_{i}|y_{j})$ is the conditional probability that $x_{i}$ occurs when $y_{j}$ occurs.

The mutual information between random variables *X* and *Y* is given as follows.

(6) $$I(X;Y)=H(X)-H(X|Y)=\sum_{i,j}p(x_{i},y_{j})\times \log_{2}\frac{p(x_{i},y_{j})}{p(x_{i})p(y_{j})}$$

## 4. Packet Loss Differentiation Model Based on Heterogeneous Ensemble Learning 

Considering the classification process, the performance of different classifiers may vary with changes in the dataset. Single classifiers often exhibit high error rates, while combining multiple classifiers can effectively reduce errors and enhance model generalization ability. Hence, this paper adopts an ensemble learning model. To ensure that individual classifiers within an ensemble classifier are “good but different”, it is common practice to employ diverse types of classifiers. Research has shown that heterogeneous classifiers tend to have higher diversity compared to homogeneous ones. Moreover, using heterogeneous ensembles can help mitigate potential biases resulting from inherent assumptions in each classification method and thus improve overall diversity. Consequently, six classification algorithms (LSSVM, DT, LR, KNN, BP neural network, and naive Bayes) were selected as initial individual classifiers for heterogeneous ensemble learning; subsequently simplified based on the principle of being “good but different”. 

In this study, the *Q*-statistic and DF value were employed to investigate the disparities among the aforementioned six individual classifiers. Let $C_{i}$ and $C_{j}$ represent two distinct classifiers. $N^{11}$ denotes the number of samples correctly classified by both classifiers, while $N^{00}$ represents the number of misclassified samples by both classifiers. Additionally, $N^{10}$ indicates instances in which $C_{i}$ was accurately classified and $C_{j}$ was misclassified. $N^{01}$ represents cases in which $C_{i}$ was incorrectly classified and $C_{j}$ was correctly classified by the classifier. The *Q*-statistic and DF value are subsequently computed using the following formulas.

(7) $$Q_{ij}=\frac{N^{11}N^{00}-N^{10}N^{01}}{N^{11}N^{00}+N^{10}N^{01}}$$

(8) $${\mathrm{DF}}_{ij}=\frac{N^{00}}{N^{11}+N^{00}+N^{10}+N^{01}}$$

According to the aforementioned equations, the *Q*-statistic value ranges from −1 to 1, while the DF value ranges from 0 to 1. A smaller value indicates a higher level of diversity in this pair of individual classifiers.

**Figure 1:**
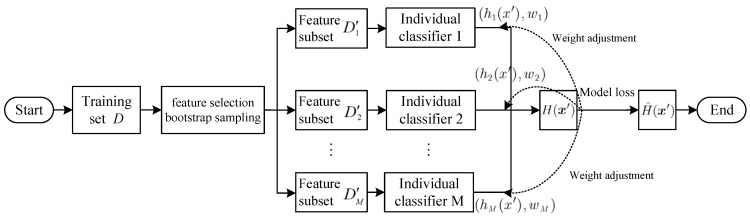
Packet loss differentiation model based on heterogeneous ensemble learning.

In order to integrate outputs from various individual classifiers within a heterogeneous ensemble learning framework and enhance learner output effectiveness in the ensemble learning algorithm, voting methods such as majority voting or weighted voting are commonly employed. The majority voting method is ineffective in utilizing the complementary information of each individual classifier. Therefore, we propose an adaptive dynamic weighted ensemble method to dynamically adjust the voting weight of each individual classifier, aiming to enhance the generalization ability of the ensemble learning algorithm for the loss cause detection model. Figure 1 illustrates the heterogeneous ensemble learning model.

Let $D^{\prime }=\{({x^{\prime }}_{i},y_{i})|{x^{\prime }}_{i}\in R^{m^{\prime }},m^{\prime }>0,y_{i}\in \{1,-1\},i=1,2,\cdots ,n^{\prime }\}$ be the subset of features generated using the feature selection algorithm, and the Sigmoid function is adopted as the default regression function for each individual classifier, i.e.,

(9) $$\ell (x^{\prime })=\frac{1}{1+e^{-\theta^{T}x^{\prime }}}$$

Suppose that the output of the *i*th individual classifier is $h_{i}(x^{\prime })$, then

(10) $$h_{i}(x^{\prime })=\left\{ \begin{array}{l} 1,\;\;\;\;\;\;\;\;\;\;\ell (x^{\prime })\;\geq 0.5\\ -1,\;\;\;\;\;\;\;\ell (x^{\prime })\;<0.5\; \end{array}\right.$$

The Sigmoid function is employed to regress the output as follows:

(11) $$p(w_{1},w_{2},\cdots ,w_{M})=\frac{1}{1+e^{-\sum_{i=1}^{M}\sum_{x^{\prime }\in {D^{\prime }}_{i}}w_{i}\;\times \;h_{i}(x^{\prime })}}$$

where $w_{i}(i=1,\cdots ,M)$ is the output weight of each individual classifier, so for any $x^{\prime }\in D$, we define:

(12) $$H(x^{\prime })=\left\{ \begin{array}{l} 1,\;\;\;\;\;\;\;\;\;\;\;\;\;p(w_{1},w_{2},\cdots ,w_{M})\geq 0.5\\ -1,\;\;\;\;\;\;\;\;\;\;p(w_{1},w_{2},\cdots ,w_{M})\;<0.5\; \end{array}\right.$$

To obtain the optimal weights $\left(w_{1},w_{2},\cdots ,w_{M}\right)$, we employ the cross-entropy loss function as the ensemble learning algorithm’s loss function, which is denoted as:

(13) $$\mathrm{Loss}=-\sum_{i=1}^{n^{\prime }}I(H({x^{\prime }}_{i})=y_{i})\log_{2}p(w_{1},\cdots ,w_{M})+I(H({x^{\prime }}_{i})\neq y_{i})\log_{2}(1-p(w_{1},\cdots ,w_{M}))$$

where I(⋅) is the indicator function, which is 1 if the condition is true and 0 if the condition is false. 

The stochastic gradient descent method is employed to dynamically adapt the voting weight vector [27] and obtain the optimal weight. The resulting output is presented as follows:

(14) $$\hat{H}(x^{\prime })=\left\{ \begin{array}{l} 1,\;\;\;\;\;\;\;\;\;\;\;\;\;p(w_{1}^{*},w_{2}^{*},\cdots ,w_{M}^{*})\geq 0.5\\ -1,\;\;\;\;\;\;\;\;\;\;p(w_{1}^{*},w_{2}^{*},\cdots ,w_{M}^{*})\;<0.5\; \end{array}\right.$$

The network packet loss discrimination algorithm proposed in this paper is referred to as the LDM-HEL algorithm. In the context of packet loss discrimination addressed in this study, channel error-induced packet loss is denoted as *W*, with samples belonging to this category represented by +1, while congestion-induced packet loss is denoted as *C*, and samples belonging to this category are represented by −1.

## 5. Simulation and Performance Analysis 

In this study, we utilized STK and NS2 to construct the simulation system for the Iridium satellite network, enabling us to simulate network packet loss scenarios and generate a comprehensive dataset. Subsequently, feature selection was performed, followed by training of a heterogeneous ensemble learning model for packet loss differentiation to validate the feasibility and effectiveness of our proposed method. Additionally, optimal parameters for relevant algorithms were determined and their rationality in terms of parameter settings is verified. Finally, in order to assess the performance advantages of our proposed method, we compared it with the LDM-satellite [11] and LLD [12] loss differentiation models under identical conditions.

### 5.1. Experimental Evaluation Indicators 

The experimental evaluation indicators in this study encompass ACCuracy (ACC), RECall (REC), PREcision (PRE), False Alarm Rate (FAR), F1-score, Area Under Curve (AUC), and Mean Absolute Error (MAE). Higher values of ACC, PRE, REC, F1-score, and AUC are indicative of superior classifier performance. The formulas for the aforementioned parameters are provided below.

$$A\mathrm{CC}=\frac{\mathrm{TP}+\mathrm{TN}}{\mathrm{TP}+\mathrm{TN}+\mathrm{FP}+\mathrm{FN}},\;\mathrm{REC}=\frac{\mathrm{TP}}{\mathrm{TP}+\mathrm{FN}},\;\mathrm{PRE}=\frac{\mathrm{TP}}{\mathrm{TP}+\mathrm{FP}},\;\mathrm{FAR}=\frac{\mathrm{FP}}{\mathrm{TN}+\mathrm{FP}},$$

$$F1-\mathrm{score}=2\times \frac{\mathrm{REC}\times \mathrm{PRE}}{\mathrm{REC}+\mathrm{PRE}},\;\mathrm{MAE}=\frac{1}{N}\times \sum_{i=1}^{N}\left|y_{i}-{\hat{y}}_{i}\right|$$

where ${\hat{y}}_{i}$ is the predicted value, $y_{i}$ is the actual value, and the parameters are from the following confusion matrix, as shown in Table 4.

The AUC represents the area under the receiver operating characteristic curve (ROC).

### 5.2. Dataset and Feature Selection Process 

**Figure 2:**
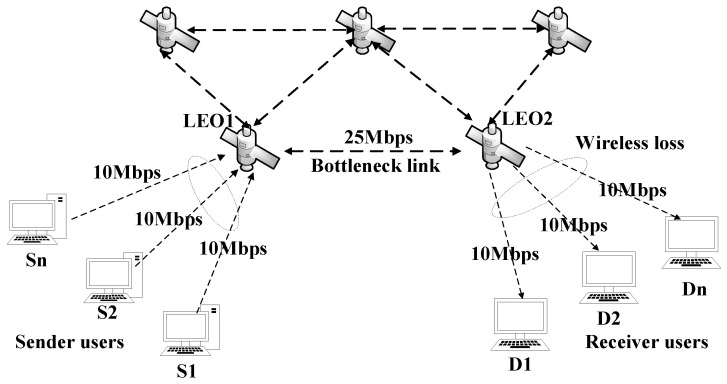
Simulation topology of satellite network.

In order to acquire packet loss data in a satellite network and subsequently evaluate the performance of loss differentiation algorithms, we employed NS2 to simulate the occurrence of packet loss in a satellite network based on the Iridium constellation. Specifically, channel error-induced packet loss was observed at the last hop of the wireless link, while congestion-induced packet loss occurred at the bottleneck link. The network topology is illustrated in Figure 2.

The network parameter configuration is as follows: The satellite-to-ground link has a bandwidth of 10 Mbps, while the inter-satellite link has a bandwidth of 25 Mbps. Background traffic consists of TCP flows generated by ON/OFF sources following a Pareto distribution with shape parameters α=1.2, and the means were, respectively, set to $\mu_{1}=500$ ms and $\mu_{2}=1300$ ms. The flow rate is set at 10 Mbps, packet size at 1000 bytes, and cache size at 50 packets. To induce congestion packet loss, initially there are two TCP flows present in the system, followed by the addition of *N* TCP flows every 10 s. Four cases are considered for *N*: *N* = 2, 4, 6, and 8. Additionally, three different bit error rates (1%, 3%, and 5%) are employed along with a tail-dropping queue management strategy to obtain the dataset on packet loss.

These data were then utilized in the Relief algorithm to derive the weight results for each feature, as presented in Table 5.

**Figure 3:**
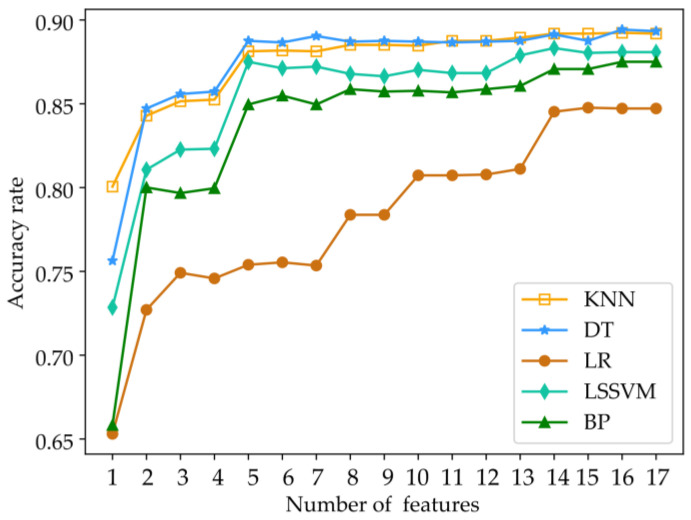
Recognition accuracy of feature subset.

The importance ranking of each feature is derived from the aforementioned table. Subsequently, based on their respective feature weights, they are sequentially incorporated into the classification model in descending order. The resulting classification accuracy is then examined. Notably, when the feature reaches approximately the 14th position, all algorithms achieve their highest accuracy levels, as depicted in Figure 3. Consequently, following the Relief algorithm-based feature selection, only the initial 14 features were retained for further analysis in this study.

The Relief algorithm is capable of ranking features, but it fails to capture the interdependence between them. In order to assess the relevance of two features in terms of their informativeness given each other, we opted for employing mutual information. Consequently, we utilized this method to investigate the correlation among 14 features selected by the Relief algorithm, and the corresponding results are presented in Table 6 below.

In the table, the bold numbers 0.96 and 1 indicate a significant correlation coefficients between $F_{8}$ and $F_{5}$ with $F_{7}$. To further streamline the features, these two features ($F_{8}$ and $F_{5}$) were eliminated in subsequent model inputs, leaving only the remaining 12 features intact.

### 5.3. Classifier Performance Analysis 

The dataset contains a total of 31,260 samples with bit error packet loss, out of which 23,267 samples were used for testing purposes. Additionally, there are 18,680 samples indicating congestion-related packet loss, among which 6000 were utilized for testing. The ensemble learning model was trained iteratively for a total of 1820 times. During the experiment, multiple independent repeated trials were conducted on the dataset and cross-validation was performed using test sets to obtain average detection results for each dataset in order to enhance the impartiality of experimental outcomes. 

To optimize the performance of the heterogeneous ensemble model, an analysis was carried out on individual learners’ predictive abilities, resulting in accuracy measures such as recall rate precision and false alarm rate being obtained for each learner (Table 7).

Table 7 reveals that the NB exhibits a low accuracy and precision rate, along with a high false alarm rate of 0.557. This indicates that when the algorithm is employed for distinguishing packet loss, a significant number of congestion loss packets are misclassified as bit error loss packets, posing substantial congestion risks to the network. Consequently, the naive Bayes method was preliminarily excluded from consideration. 

Moreover, in order to achieve more accurate prediction results, it becomes imperative to select individual classifiers with greater diversity. In this study, we utilized Q statistic value and DF value to quantify the dissimilarity among individual classifiers (as presented in Table 8 and Table 9).

Furthermore, it can be observed from Table 8 that the Q statistic value of the BP neural network is 0.9, ranking second-highest. Table 9 indicates that the DF value of the BP neural network is 0.128, which is the highest among all classifiers considered. Additionally, considering algorithm complexity and false alarm rate, the BP neural network does not perform optimally in discriminating data packet loss; hence, it was excluded from the final selection scheme for individual classifiers, leaving four types: LSSVM, DT, LR, and kNN. 

**Figure 4:**
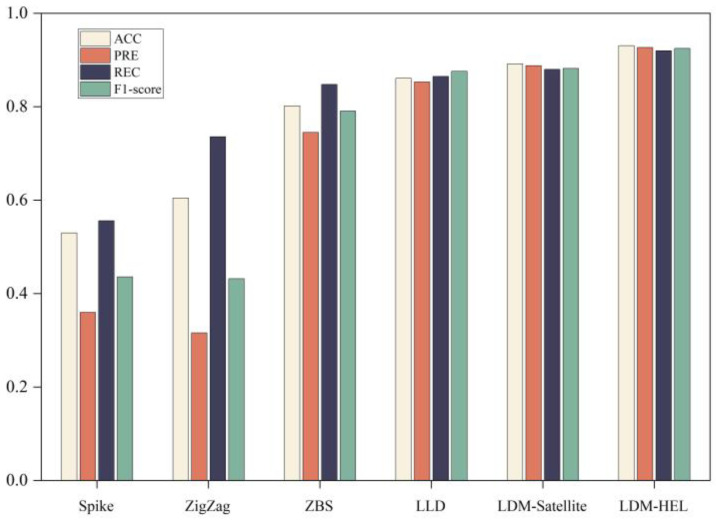
Performance comparison between proposed LDM-HEL and the other five classifiers.

To validate the performance enhancement of our proposed integrated detection method LDM-HEL compared to existing methods such as LDM-satellite [11], LLD [12], Spike method, Zigzag method, and ZBS method [4,5,6]; we plotted average performance results for four detection indicators (accuracy, precision, recall, and F1-score) across five different detection methods as shown in Figure 4.

Firstly, as depicted in Figure 4, the proposed LDM-HEL, the LDM-Satellite, and the LLD method have shown significant performance enhancements compared to conventional classifiers. Moreover, the accuracy, precision, recall, and F1-score metrics of the proposed heterogeneous ensemble method surpass those of the other five classifiers, indicating its superior comprehensive detection performance and low false negative rate in effectively identifying bit errors and packet losses. 

Analyzing the causes of the performance difference, it was found that the Spike schemes only consider one packet loss feature and need to set a threshold to determine the packet loss type. The setting of the threshold is related to the characteristics of the network and the experience of the manager, which affects the packet loss classification performance. The ZigZag and ZBS schemes consider multi-features, and the packet loss differentiation ability is improved, but its threshold also needs to be set reasonably. The LDM-satellite algorithm has better performance, but it uses a homogeneous integrated model, and its classification accuracy is not optimal. RTT is employed in the LLD algorithm. The change of RTT in the low-orbit satellite network not only reflects the network congestion, but also may be caused by the change of the distance between nodes, so it is easy to cause packet loss and misjudgment. Due to the heterogeneous individual classification model and the integration strategy based on stochastic gradient descent, the accuracy rate, accuracy rate, recall rate, and F1-score indexes of the proposed algorithm are higher than those of the other five classifiers, which indicates that the proposed heterogeneous integration detection method has good comprehensive detection performance and low false negatives, and can effectively detect packet loss caused by channel error.

**Figure 5:**
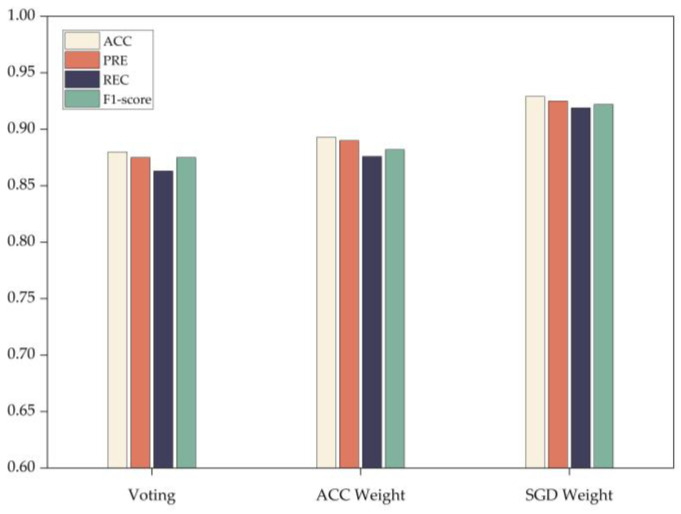
Performance comparison of the three weighting algorithms.

Furthermore, employing the heterogeneous integration architecture proposed in this paper and adjusting the approach for generating final integration results by utilizing the voting method, ACC weighting method based on accuracy assessment, as well as the SGD-based weighting method for comparison purposes (as depicted in Figure 5), reveals that the SGD-based weighting significantly enhances accuracy, precision, recall, and F1-score measurements. This enhancement is attributed to the dynamic adaptive weighted integration method introduced in the integration process, which dynamically adjusts the voting weight of each base classifier, thereby improving the generalization ability of the multi-classifier integrated detection model.

**Figure 6:**
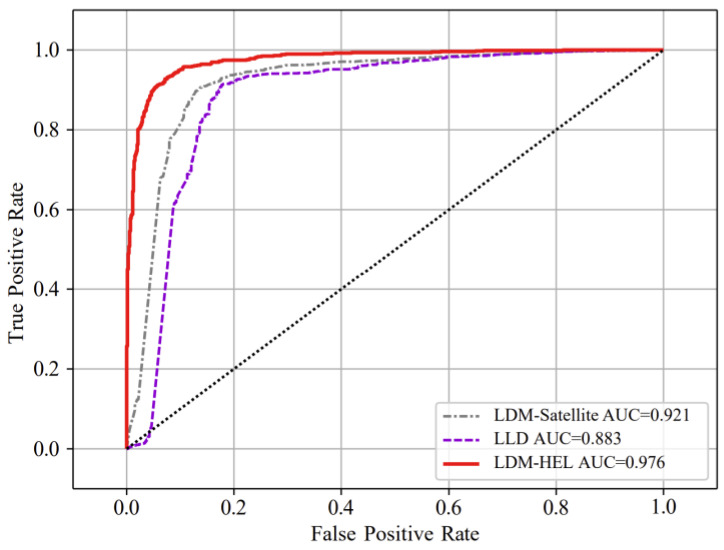
Comparison of AUC values of LDM-HEL, LDM-satellite, and LLD algorithms.

The AUC values were calculated and compared with the LDM-satellite and LLD methods to validate the comprehensive advantages of the proposed method in detecting packet loss differentiation performance. As depicted in Figure 6, the AUC value of the proposed method is 0.976, surpassing that of the other two methods (0.883 and 0.921), thereby indicating its superior overall efficacy.

**Figure 7:**
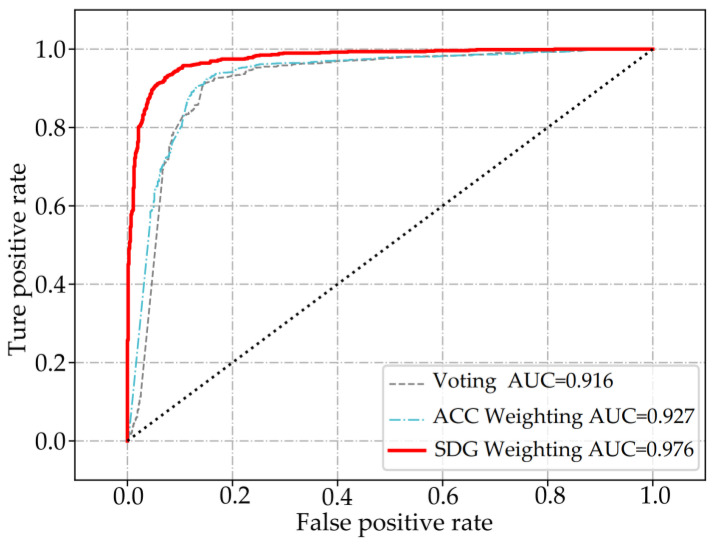
Comparison of AUC values of voting, ACC weighting, and SDG weighting.

Moreover, the proposed heterogeneous integration architecture is utilized and the resulting integrations are combined using a voting method, ACC weighting method, and SGD-based weighting method, respectively. The ROC graph in Figure 7 illustrates that the AUC value of the SGD-based weighting method (0.976) surpasses those of the other two methods (0.927 and 0.916), indicating its superiority.

**Figure 8:**
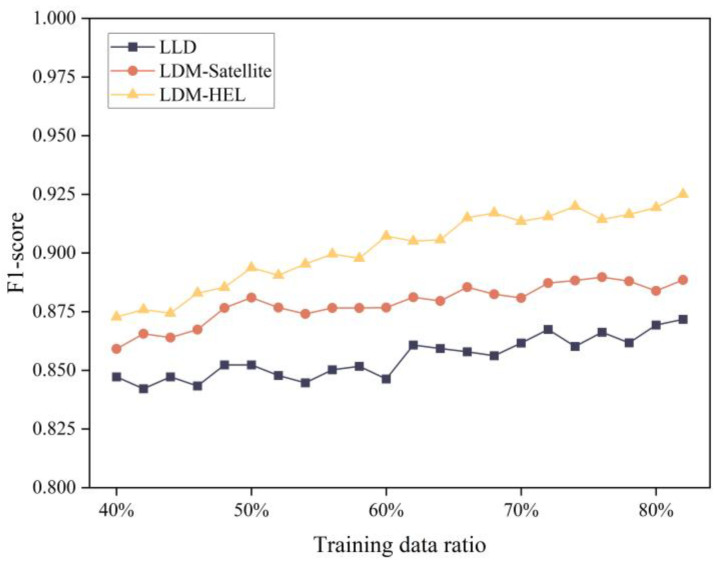
Comparison of F1-score performance of three packet loss differentiation models.

**Figure 9:**
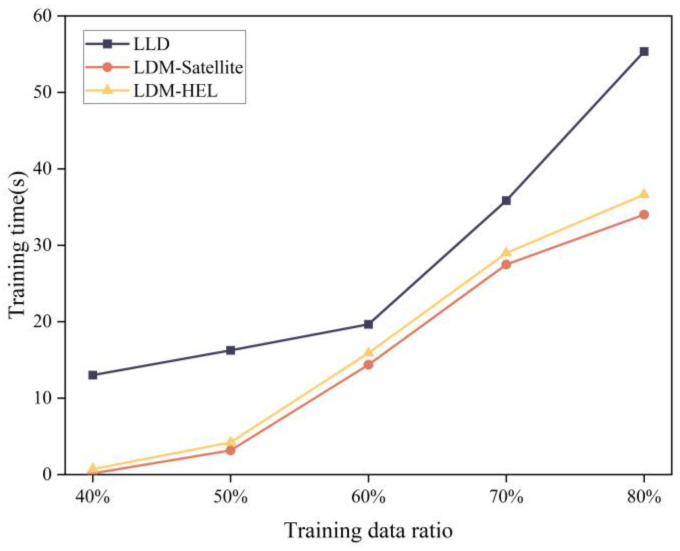
Comparison of training time of three kinds of packet loss differentiation models.

In order to verify the performance of the proposed method compared with the existing learning model-based packet loss discrimination methods, this experiment uses the same sampled data set to detect packet loss differentiation. The LLD, LDM-satellite, and LDM-HEL methods were selected to assess the F1-score performance and training time of different differentiation methods under various training data ratios. As shown in Figure 8, among the three differentiation methods, the LDM-satellite and LDM-HEL methods show a gradual increase with the growth of the training data ratio, and generally, the F1-score of our LDM-HEL method is superior to the other two methods. Additionally, the comparison of training time is presented in Figure 9. The training time of the three packet loss differentiation methods increases with the rise in the training data ratio. The training time of the proposed method is shorter than LLR and slightly higher than LDM-satellite.

### 5.4. Network Performance Analysis

The packet loss differentiation algorithm proposed in this paper requires the cooperation of the receiver because it uses information such as ROTT and IAT at the receiver. In other words, the final packet loss differentiation is completed at the receiver, necessitating the receiver to provide feedback on the judgment information to the sender. To achieve this, the explicit loss notification (ELN) mechanism is employed. When the receiver determines that the cause of packet loss is congestion loss, the ELN flag bit of the TCP packet header is set to 0, and when the receiver determines that the cause of packet loss is bit error loss, the ELN flag bit of the TCP packet header is set to 1. Accordingly, the sender adopts the corresponding congestion control window adjustment strategy.

**Figure 10:**
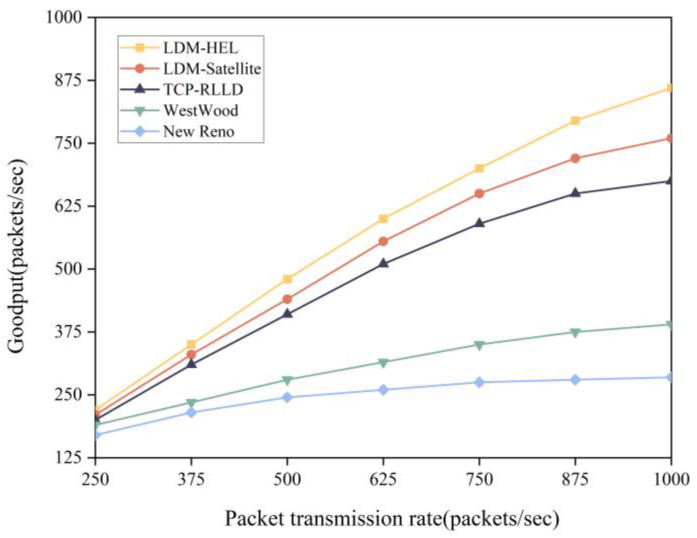
Comparison of Goodput rates between LDM-HEL, LDM-satellite, TCP-RLLD, Westwood, and New Reno.

In the experiment, the link bit error rate was set to 10^−1^, and LDM-HEL, LDM-satellite, and TCP-RLLD [26] packet loss discrimination algorithms were applied to TCP New Reno. We evaluated the Goodput performance of the LDM-HEL algorithm and four baseline algorithms (LDM-Satellite, TCP-RLLD, New Reno, Westwood, LA, USA) at different end-to-end packet sending rates, as shown in Figure 10.

The experimental results show that New Reno and Westwood do not exhibit significant Goodput improvement when the transmission rate increases. This is mainly due to their lack of an effective packet loss differentiation mechanism. In contrast, LDM-HEL demonstrates better performance. By employing a heterogeneous ensemble learning model, it comprehensively considers more network state characteristics, fully leverages the advantages of multiple classifiers, and enhances the model’s generalization ability. This results in more accurate packet loss differentiation, helping to avoid unnecessary congestion window reduction. Compared with the LDM-satellite and the TCP-RLLD algorithms, its throughput increased by an average of 9.28% and 19.73%, respectively.

**Figure 11:**
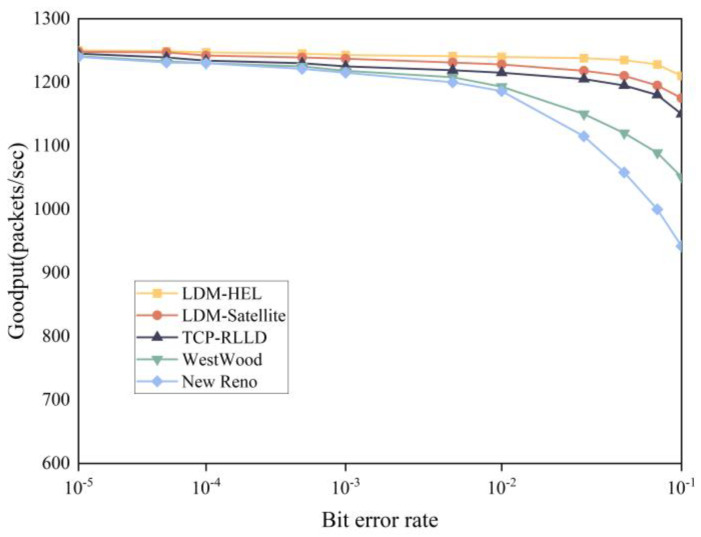
Comparison of Goodput with different protocols for LEO.

We also compare the performance of each algorithm under different bit error rates, and the results are shown in Figure 11. We set the packet sending rate to 1250 packets/sec. In the case of a low packet loss rate, the Goodput of all protocols is relatively high. With the increase in bit error rate, the Goodput of each algorithm decreases obviously. However, LDM-HEL still exhibits a better Goodput.

## 6. Conclusions

This paper proposes a packet loss differentiation method based on heterogeneous ensemble learning, aiming to address the high bit error characteristics of satellite networks and the inability of traditional TCP protocols to differentiate between bit error loss and congestion loss. Firstly, we summarize the packet features used in existing packet loss differentiation algorithms, including ROTT, IAT, and the number of consecutive packet losses. Relief and mutual information algorithms are employed for feature selection. Then, a heterogeneous ensemble consisting of LSSVM, DT, LR, and kNN is utilized. In order to enhance the generalization ability of the heterogeneous ensemble learning algorithm, an adaptive dynamic weighted ensemble method is introduced to adjust the voting weight of each individual classifier. Simulation results demonstrate that LDM-HEL achieves higher accuracy and significantly improves throughput performance when applied to TCP.

In the future, we will apply LDM-HEL to real network traffic data for further evaluation. LDM-HEL can also be applied to other wireless networks or wired/wireless hybrid networks to distinguish packet loss, but it needs to select suitable packet features and effective individual classifiers according to network characteristics. In addition to link errors and congestion, packet loss caused by routing is also a problem worth studying. Finally, LDM-HEL is a packet loss discrimination method, and the next step is to improve the accuracy of packet loss discrimination by combining cross-layer information.

## Figures and Tables

**Table 1:** The features regarding the end-to-end ROTT.

Feature Number	Symbol	Interpretation
$F_{1}$	ROTT	The one-way trip time of the last normal received packet
$F_{2}$	${\mathrm{ROTT}}_{\min}$	Minimum end-to-end ROTT from the last packet loss to the present packet
$F_{3}$	${\mathrm{ROTT}}_{\mathrm{mean}}$	Average end-to-end ROTT from the last packet loss to the present packet
$F_{4}$	${\mathrm{ROTT}}_{\mathrm{dev}}$	End-to-end ROTT deviation from the last packet loss to the present packet
$F_{5}$	$\frac{\mathrm{ROTT}}{{\mathrm{ROTT}}_{\mathrm{mean}}}$	The ratio of the ROTT of the last normal received packet to the average ROTT
$F_{6}$	$\frac{\mathrm{ROTT}}{{\mathrm{ROTT}}_{\min}}$	The ratio of the ROTT of the last normal received packet to the minimum ROTT
$F_{7}$	$\frac{{\mathrm{ROTT}}_{\min}}{{\mathrm{ROTT}}_{\mathrm{mean}}}$	The ratio of the minimum ROTT to the average ROTT
$F_{8}$	$\frac{\mathrm{ROTT}}{{\mathrm{ROTT}}_{\mathrm{mean}}-{\mathrm{ROTT}}_{\mathrm{dev}}}$	The ratio of the ROTT to the difference between the mean and deviation of the last normal received packet

**Table 2:** The features regarding IAT.

Feature Number	Symbol	Interpretation
$F_{9}$	IAT	The interval of the last pair of adjacent packets
$F_{10}$	${\mathrm{IAT}}_{\min}$	The minimum interval between the last packet loss and the present packet loss
$F_{11}$	${\mathrm{IAT}}_{\mathrm{mean}}$	The average interval between the last packet loss and the present packet loss
$F_{12}$	${\mathrm{IAT}}_{\max}$	The maximum interval between the last packet loss and the present packet loss
$F_{13}$	$\frac{\mathrm{IAT}}{{\mathrm{IAT}}_{\mathrm{mean}}}$	The ratio of the IAT to the average IAT
$F_{14}$	$\frac{\mathrm{IAT}}{{\mathrm{IAT}}_{\min}}$	The ratio of the IAT to the minimum IAT
$F_{15}$	$\frac{\mathrm{IAT}}{{\mathrm{IAT}}_{\max}}$	The ratio of the IAT to the maximum IAT
$F_{16}$	$\frac{\mathrm{IAT}(t)}{\mathrm{IAT}(t-1)}$	The ratio of the interval between the last pair of adjacent packets to the previous pair of adjacent packets

**Table 3:** Packet loss number.

Feature Number	Symbol	Interpretation
$F_{17}$	${\mathrm{NUM}}_{\mathrm{loss}}$	Number of consecutive lost packets

**Table 4:** Confusion matrix.

Confusion Matrix		True Class	
		Positive	Negative
Predicting classes	Positive	True Positive (TP)	False Positive (FP)
	Negative	False Negative (FN)	True Negative (TN)

**Table 5:** Feature weight calculation results.

No.	Symbol	Feature Number	Feature Weight
1	${\mathrm{IAT}}_{\mathrm{mean}}$	$F_{11}$	0.031094
2	ROTT	$F_{1}$	0.022750
3	${\mathrm{ROTT}}_{\min}/{\mathrm{ROTT}}_{\mathrm{mean}}$	$F_{7}$	0.017432
4	${\mathrm{IAT}}_{\max}$	$F_{12}$	0.006748
5	IAT(t)/IAT(t−1)	$F_{16}$	−0.007490
6	$\mathrm{ROTT}/{\mathrm{ROTT}}_{\min}$	$F_{6}$	−0.009605
7	${\mathrm{ROTT}}_{\mathrm{dev}}$	$F_{4}$	−0.009641
8	${\mathrm{NUM}}_{\mathrm{loss}}$	$F_{17}$	−0.010733
9	$\mathrm{ROTT}/({\mathrm{ROTT}}_{\mathrm{mean}}-{\mathrm{ROTT}}_{\mathrm{dev}})$	$F_{8}$	−0.012359
10	$\mathrm{ROTT}/{\mathrm{ROTT}}_{\mathrm{mean}}$	$F_{5}$	−0.012709
11	${\mathrm{ROTT}}_{\mathrm{mean}}$	$F_{3}$	−0.013706
12	${\mathrm{ROTT}}_{\min}$	$F_{2}$	−0.026769
13	$\mathrm{IAT}/{\mathrm{IAT}}_{\min}$	$F_{14}$	−0.094195
14	$\mathrm{IAT}/{\mathrm{IAT}}_{\mathrm{mean}}$	$F_{13}$	−0.111619
15	${\mathrm{IAT}}_{\min}$	$F_{10}$	−0.235939
16	IAT	$F_{9}$	−0.323253
17	$\mathrm{IAT}/{\mathrm{IAT}}_{\max}$	$F_{15}$	−0.628012

**Table 6:** Feature mutual information matrix.

	$F_{11}$	$F_{1}$	$F_{7}$	$F_{12}$	$F_{16}$	$F_{6}$	$F_{4}$	$F_{17}$	$F_{8}$	$F_{5}$	$F_{3}$	$F_{2}$	$F_{14}$	$F_{13}$
$F_{11}$	1	0.34	0.75	0.14	0.45	0.34	0.70	0.08	0.79	0.79	0.62	0.03	0.32	0.93
$F_{1}$	0.34	1	0.40	0.10	0.40	0.90	0.50	0.06	0.48	0.48	0.34	0.06	0.15	0.35
$F_{7}$	0.75	0.40	1	0.09	0.34	0.41	0.88	0.09	**0.96**	**0.96**	0.88	0.04	0.30	0.78
$F_{12}$	0.14	0.10	0.09	1	0.13	0.10	0.10	0.02	0.10	0.10	0.11	0.22	0.05	0.05
$F_{16}$	0.45	0.40	0.34	0.13	1	0.41	0.37	0.06	0.40	0.40	0.26	0.02	0.37	0.50
$F_{6}$	0.34	0.90	0.41	0.10	0.41	1	0.51	0.06	0.49	0.49	0.35	0.07	0.15	0.36
$F_{4}$	0.70	0.50	0.88	0.10	0.37	0.51	1	0.08	0.92	0.92	0.89	0.03	0.31	0.73
$F_{17}$	0.08	0.06	0.09	0.02	0.06	0.06	0.08	1	0.11	0.11	0.04	0.02	0.03	0.10
$F_{8}$	0.79	0.48	0.96	0.10	0.40	0.49	0.92	0.11	1	**1.00**	0.85	0.04	0.35	0.82
$F_{5}$	0.79	0.48	0.96	0.10	0.40	0.49	0.92	0.11	1.00	1	0.85	0.04	0.35	0.82
$F_{3}$	0.62	0.34	0.88	0.11	0.26	0.35	0.89	0.04	0.85	0.85	1	0.04	0.22	0.65
$F_{2}$	0.03	0.06	0.04	0.22	0.02	0.07	0.03	0.02	0.04	0.04	0.04	1	0.01	0.01
$F_{14}$	0.32	0.15	0.30	0.05	0.37	0.15	0.31	0.03	0.35	0.35	0.22	0.01	1	0.40
$F_{13}$	0.93	0.35	0.78	0.05	0.50	0.36	0.73	0.10	0.82	0.82	0.65	0.01	0.40	1

**Table 7:** Hyperparameters of individual learners and their performance in the dataset.

Individual Learner	Hyperparameters	ACC	REC	PRE	FAR
LSSVM	Punishment coefficients are 10, γ=0.4; the kernel function is the radial basis kernel function	0.821	0.795	0.816	0.245
KNN	The number of neighbors is 7	0.887	0.874	0.882	0.184
LR	Type of regularization is L2, the inverse of the regularization strength is 1000, the type of the solver is Liblinear	0.775	0.730	0.778	0.392
DT	The split node criteria are information entropy, the maximum depth of the tree is 11, the minimum number of samples needed at leaf nodes is 3, the minimum number of samples to split an internal node is 29, and the split strategy at the node is best	0.869	0.855	0.864	0.173
BP neural network	1 input layer, 1 hidden layer, 30 nodes, Relu activation function, 1 output layer, using Sigmoid activation function, using binary_crossentropy as the loss funQction and Adam Optimizer	0.823	0.832	0.803	0.327
Naive Bayes	None	0.743	0.841	0.732	0.557

**Table 8:** Q statistics among individual classifiers.

Individual Classifier	Q Statistics					
	DT	LR	kNN	BP	Mean	System
LSSVM	0.764	0.985	0.886	0.997	0.908	
DT		0.699	0.954	0.735	0.788	
LR			0.850	0.996	0.882	0.874
kNN				0.873	0.891	
BP					0.900	

**Table 9:** DF values among individual classifiers.

Individual Classifier	DF Values					
	DT	LR	kNN	BP	Mean	System
LSSVM	0.068	0.161	0.077	0.172	0.119	
DT		0.069	0.081	0.071	0.072	
LR			0.078	0.191	0.125	0.105
kNN				0.080	0.079	
BP					0.128	

## Data Availability

Data are contained within the article.
